# Intelligent DDoS Attack Detection in Software-Defined Networks Using Explainable Machine Learning

**DOI:** 10.3390/s26175610

**Published:** 2026-09-03

**Authors:** Javaid Ahmad Malik, Naila Samar Naz, Muhammad Saleem, Muhammad Adnan Khan

**Affiliations:** 1 Department of Computer Science, National College of Business Administration and Economics, Lahore 54000, Pakistan; 2203101@ncbae.edu.pk (J.A.M.); nailanaz@ncbae.edu.pk (N.S.N.); msaleem@ncbae.edu.pk (M.S.); 2 Department of Software, Faculty of Artificial Intelligence and Software, Gachon University, Seongnam-si 13557, Republic of Korea

**Keywords:** Software-Defined Networking (SDN), machine learning (ML), Explainable Artificial Intelligence (XAI)

## Abstract

The recent trend of Software-Defined Networking (SDN) has posed significant cybersecurity challenges as a result of its centralized control architecture, dynamic traffic behavior, and high programmability. Although these attributes improve network flexibility and management, they also increase vulnerability to Distributed Denial-of-Service (DDoS) attacks that can overwhelm network resources and disrupt services. Traditional signature- and rule-based detection methods may struggle with evolving traffic patterns and generate excessive false alarms. Machine learning offers a more promising solution that can learn the complex traffic patterns and separate malicious traffic from normal traffic. Most machine learning models, however, are black-box models that provide only superficial insight into the model predictions. Explainable Artificial Intelligence (XAI) addresses this limitation by identifying influential traffic features and providing interpretable evidence for detection decisions. This research develops an explainable machine learning-based framework for accurate, transparent, and reliable DDoS attack detection in an SDN environment. Several machine learning models are assessed, and XAI techniques are applied to explain the results of the predictions at global and instance levels. Gradient Boosting, Logistic Regression, AdaBoost, and Gaussian Naive Bayes were evaluated on 104,345 network-flow records using a 70:30 training–testing split. Gradient Boosting achieved the strongest performance, with 99.88% training accuracy, 99.87% testing accuracy, a testing F1-score of 99.84%, and a 0.20% miss rate. SHAP identified the most influential traffic features, while LIME linked individual predictions to feature-specific contributions. The proposed framework therefore combines reliable DDoS detection with transparent, analyst-oriented decision support for SDN security monitoring.

## 1. Introduction

With the widespread adoption of cloud computing, Internet-based services and inter-connected digital infrastructures, organizations have become far more reliant on communication networks that provide security, scalability, and reliability. In this changing landscape, Software-Defined Networking (SDN) has become a new paradigm for networking that separates the control plane from the data plane, which allows for central intelligence of the network, dynamic programmability, and intelligent traffic management [1,2]. Unlike traditional networking architectures, SDN gives greater flexibility, scalability, and operational efficiency because it enables network policies and forwarding rules to be set using software-based controllers. As a result, these capabilities have enabled its widespread adoption in cloud-based deployments, enterprise networks, data centers, Internet of Things deployments, and other large-scale distributed systems. However, the centralized, programmable nature of SDN poses serious security risks as well. Multiple network components can be impacted by a compromised controller, malicious application, or manipulated communication channel, which makes SDN infrastructures good targets for sophisticated cyberattacks.

Such threats span the application, control, and data planes and may involve unauthorized policy injection, flow-rule manipulation, and controller or flow-table exhaustion. Recent work employing a feedforward neural network optimized through Artificial Bee Colony optimization demonstrated effective detection of known and novel intrusions, supporting adaptive ML-based protection for dynamic networks [3].

Ransomware is one of the most devastating and costliest ransom-type malware threats in today’s network environment. Ransomware generally denies access to systems and/or encrypts valuable data and then demands payment from victims to restore access [4]. In addition to data encryption, modern ransomware variants could steal credentials, move laterally, exfiltrate data, escalate privileges, and disrupt services prior to the final ransomware stage. These attacks can be devastating to business continuity, inflict damage on confidential data, harm organizations’ images, and save a significant amount of money in recovery expenses. In centralized, highly programmable environments like SDN, ransomware has the potential to spread quickly and easily between interconnected devices, making its impact even more dire in these scenarios.

Traditional DDoS attack-detection methods are largely based on predefined ransomware signatures, static ransomware rules, manual configuration of traffic thresholds, and known indicators of compromise [5,6]. While these methods can detect DDoS attack types, they are not very effective against zero-day attacks, polymorphic ransomware, encrypted communications, obfuscated traffic, and continually evolving ransomware attack strategies. The signature-based approach generally needs to be based on a known pattern of malicious traffic, and rule-based methods can create excessive false alarms if network traffic behavior is dynamic. The restrictions imposed by these limitations are not well suited for today’s SDN environments where traffic characteristics and application needs can shift at will, and network policies need to be flexible.

Centralized visibility and programmability of SDN offer an opportunity to tackle these challenges more effectively. SDN controllers gather the overall traffic statistics, track the flow of communications, and dynamically enforce security policies throughout the network. To this end, SDN-based security frameworks that rely on observations at the controller level to detect suspicious communication patterns, isolate infected devices, and prevent propagation of ransomware attacks have been investigated [7]. However, manual analysis is challenging due to the large volume, high dimensionality, and dynamics of SDN traffic. Furthermore, the fixed security rules can miss out on complex and unknown network-flow patterns and relationships with ransomware behavior.

Machine Learning (ML) has therefore gained significant attention as an intelligent approach for identifying malicious network activities. ML algorithms can process large volumes of traffic data, learn complex behavioral relationships, and distinguish DDoS attack flows from legitimate communication patterns. By examining statistical and temporal network-flow characteristics, ML models can detect subtle deviations that may not be captured through static signatures or manually defined rules. Previous studies have demonstrated the effectiveness of ML techniques in identifying ransomware activities using traffic-flow features and behavioral indicators [8]. Similarly, ML-based intrusion detection systems have achieved improved detection capability and adaptability compared with conventional rule-based and signature-based mechanisms in SDN environments [9].

Despite their strong predictive performance, many advanced ML models operate as black-box systems whose internal decision-making processes are difficult to understand. A model may classify a network flow as ransomware-related without clearly explaining which features influenced the prediction or why the traffic was considered malicious. This lack of transparency presents a major limitation in cybersecurity applications, where detection outcomes may trigger critical actions such as traffic blocking, device isolation, service suspension, or incident escalation. Security analysts require sufficient evidence to validate model decisions, investigate suspicious behavior, and avoid unnecessary disruption caused by false alarms. Therefore, predictive accuracy alone is insufficient for the practical deployment of intelligent DDoS attack-detection systems.

Explainable Artificial Intelligence (XAI) has emerged as an important research direction for addressing the interpretability limitations of black-box ML models. XAI techniques aim to provide human-understandable explanations by identifying influential input features, quantifying their contribution to individual predictions, and revealing the broader behavior of a trained model [10]. In the context of cybersecurity, such explanations can help analysts determine whether a prediction is based on meaningful attack indicators or irrelevant correlations. The integration of XAI with ML-based intrusion detection systems can consequently support both accurate threat identification and transparent decision analysis [11]. Global explanations can reveal the overall features most strongly associated with ransomware activity, whereas local explanations can clarify why a specific traffic instance has been classified as malicious or legitimate.

However, the integration of explainability into DDoS attack detection for SDN remains insufficiently explored. Existing studies often emphasize detection accuracy while providing limited analysis of model transparency, feature influence, and decision justification. In addition, ransomware behavior continues to evolve through advanced encryption mechanisms, covert communication channels, fileless execution, traffic obfuscation, and adaptive evasion strategies. These developments create a need for intelligent detection mechanisms that can identify complex attack patterns while also providing transparent and reliable reasoning for their predictions [12]. Without such explanations, network administrators may be unable to confidently validate alerts or translate model outputs into timely security responses.

To address these limitations, this study proposes an explainable machine learning-based framework for accurate, transparent, and reliable DDoS attack detection in SDN environments. The proposed approach analyzes network-flow characteristics using multiple ML classifiers and integrates XAI techniques to interpret their prediction outcomes. The framework is designed not only to determine whether network traffic is legitimate or DDoS-related but also to identify the features that contribute most strongly to each decision. In this way, it combines the predictive capability of data-driven models with the transparency required for practical cybersecurity operations.

Gradient Boosting, SHAP, and LIME are established techniques; therefore, this study does not claim novelty in the individual algorithms. Instead, its contribution lies in their integration within a unified SDN flow-analysis workflow that combines multi-model comparison, multi-metric model selection, and complementary global and instance-level explanations.

The major contributions of this study are summarized as follows:

- A unified explainable ML framework is developed for DDoS attack detection in SDN using network-flow features and multiple supervised classifiers.
- The most reliable DDoS detector is selected through a consistent comparative evaluation using accuracy, precision, recall, specificity, F1-score, ROC-AUC, MCC, and miss rate.
- Global SHAP and local LIMEs are jointly applied to the selected DDoS detection model to identify influential traffic features and explain individual benign and attack predictions.

The remainder of this paper is organized as follows. Section 2 reviews the relevant literature on DDoS attack detection, ML-based intrusion detection, SDN security, and XAI-enabled cybersecurity frameworks. Section 4 presents the proposed methodology, including data preparation, model development, evaluation measures, and explanation mechanisms. Section 5 discusses the experimental results and interpretation outcomes. Finally, Section 6 concludes the study and identifies potential directions for future research.

## 2. Literature Review

Multiple studies have investigated ransomware and network intrusion detection using ML-, DL-, and SDN-based approaches. The following literature critically reviews these studies in terms of their objectives, adopted methods, key achievements, and remaining limitations. In [13], the authors focused on DDoS attack detection when the network packet is encrypted while transported through an HTTPS connection, in which deep packet inspection is not effective. They suggested an SDN-based framework in which they extracted features from the flow level by using Programmable Forwarding Engines and classified traffic with a Random Forest model. The approach successfully detected more than 0.86 with a false-negative rate below 0.11, proving the feasibility of early detection of ransomware without inspecting the payload. The use of one classifier, the number of missed attacks and the lack of interpretability of the model, however, made it less reliable.

In [14], the researchers reviewed context-aware and anomaly-based DDoS attack-detection studies published between 2020 and 2024, emphasizing the limitations of signature-based methods against novel and polymorphic attacks. The review examined techniques such as dynamic entropy profiling, behavioral mapping, machine learning, deep learning, and federated learning. It highlighted improved adaptability and privacy-preserving detection but identified persistent challenges involving limited and imbalanced datasets, complex data fusion, high computational cost, and insufficient real-time capability. The study consequently emphasized explainability, rapid detection, and privacy preservation as key directions for future ransomware defense.

The researchers in [15] conducted a survey of the increasingly complex DDoS attack detection using deep learning techniques, such as cryptoviral and zero-day variants. The study analyzed the current deep learning models and pointed out their increasing importance for detecting complex malicious patterns that cannot be detected by traditional detection approaches. It also stressed the overall importance of machine learning in detecting malware, fraud analysis, spoofing detection and cybersecurity risk assessments. However, the review revealed that existing methods still had some drawbacks, such as poor generalization across unseen families, high computational cost, lack of interpretability and the lack of more capable real-time detection methods.

In [16], they examined how ransomware is detected using ML and DL, highlighting the devastating effects of ransomware, the complexity of ransomware reversement and the importance of identifying ransomware before a system is compromised. The study reviews the most prominent approaches that have the ability to learn ransomware behavior and detect zero-day variants, as well as experimentally explores their resistance to the changing ransomware. It found that generalization, adaptability, and early detection are still challenges, though, especially as ransomware increasingly attacks IoT environments.

Zhang et al. [17] summarized the recent developments in the field of ML-based intrusion detection and highlighted the limitations of signature-based IDS. Considering the fact that signature-based IDS cannot detect the new and sophisticated types of cyber-attacks, Zhang et al. [17] reviewed the recent advancements in the field of ML-based IDS. The study covered the major developments such as zero-day attack detection, encrypted traffic analysis, XAI integration, graph neural networks and large language models for contextual traffic interpretation. These methods enable adaptability, transparency and detection, but there are yet unresolved issues such as computational complexity, trustworthiness of models, scaling, data quality and reliable deployment in dynamic network environments, which the authors pointed out.

To overcome this problem of deteriorating performance of conventional IDS against the complex cyber-attacks, the researchers in [18] discussed the use of AI in intrusion detection. The study included ML and DL models like SVM, Random Forest, CNN and GAN as well as popular tools like Suricata, Zeek and Snort. The above approaches were judged on the basis of accuracy, false-positive rate, scalability, and computational efficiency. The review also pointed to new trends like XAI, federated learning, and hybrid IDS. But, dataset bias, adversarial robustness, computational costs, deployment complexity and model selection are still a major challenge in cloud, IoT and enterprise-use cases.

To overcome the increasing number and complexity of network attacks, the researchers in [19] conducted a survey of the different approaches to intrusion detection that have been proposed in the last decade based on ML. The study compared signature-based systems that only recognize known attacks with anomaly-based systems that employ ML to differentiate between normal and malicious activity, creating a defense against attacks that are not in the signature database. While ML-based intrusion detection displayed high adaptability, the survey pointed to ongoing challenges with false alarms, limited datasets, model generalization, scalability and deployment to the real world.

The researchers in [20] surveyed recent network intrusion detection systems (NIDS) based on machine learning, emphasizing the limitations in the datasets, feature-level processing, and supervised multi-class classification. The study was a comparison of the widely used datasets, such as UNSW-NB15, TUIDS and NSL-KDD, and a presentation of preprocessing techniques to improve the detection performance. It also identified ongoing issues with outdated and imbalanced data sources, redundant features, poor model generalizability, and real-world deployment in a changing network environment.

Addressing the difficulty of analyzing large and unpredictable network traffic, one study [21] proposed an ML-based intrusion detection framework for identifying emerging attacks beyond the capability of signature-based systems. PCA was applied for dimensionality reduction, while KNN, Random Forest, Decision Tree, and SVM were used to classify traffic based on flow and connection features. The framework achieved high detection accuracy and supported real-time monitoring through a Flask-based interface. However, its reliance on conventional classifiers, limited discussion of dataset diversity, and absence of explainability analysis may restrict its generalization and analyst trust.

Ashiku and Dagli [22] developed a CNN-based network intrusion detection system with semi-dynamic hyperparameter tuning for ten-class classification on the UNSW-NB15 dataset, achieving up to 95.6% accuracy. However, class imbalance reduced the detection of underrepresented attacks, while SDN-specific deployment and model explainability were not evaluated.

Table 1 compares existing ransomware and intrusion detection studies in terms of methods, objectives, achievements, explainability, strengths, and limitations. Recent ransomware detection mechanisms primarily include network-flow-based ML classification, SDN-controller-based packet and SMB inspection, automated traffic blocking, honeypot monitoring, hybrid static–dynamic analysis, and XAI-supported behavioral detection [13,14,15,16]. Most studies employed ML-, DL-, or SDN-based techniques and demonstrated improved detection of known, unknown, or encrypted threats [13,14,15,16,17,18,19,20,21]. However, several approaches were limited by poor generalization, dataset imbalance, computational complexity, false alarms, and a lack of explainable decision-making. In contrast, the proposed work combines multiple ML models with XAI to achieve both high detection performance and transparent ransomware classification in SDN environments.

## 3. Gap Analysis

The comparative findings in Table 1 show that existing ransomware and intrusion detection studies remain limited in several important areas. Although many approaches report strong detection performance, they often rely on isolated models, non-transparent decision processes, or limited experimental settings [13,19,20,21]. Review studies also highlight persistent concerns related to dataset imbalance, weak generalization, computational complexity, scalability, and real-time deployment [14,15,16,17,18,19,20]. The key research gaps are summarized as follows:

- **Limited ransomware-specific SDN frameworks:** Most studies focus on general intrusion detection rather than ransomware behavior in SDN environments [17,18,19,20,21].
- **Insufficient model transparency:** Several approaches provide accurate predictions but do not explain the traffic features responsible for detection decisions [13,15,16,19,20,21].
- **Restricted comparative evaluation:** Existing studies rarely combine multiple supervised models, XAI, high detection reliability, and analyst-oriented explanations in one framework.

To address these gaps, the proposed study develops an explainable ML-based DDoS attack-detection framework specifically for SDN environments. Multiple supervised models are evaluated, while XAI is integrated to identify influential traffic features and justify individual predictions. Gradient Boosting achieves 99.72% training accuracy and 99.65% testing accuracy, along with perfect precision, recall, and F1-score values. Thus, the proposed framework improves detection reliability, transparency, analyst trust, and practical decision support for intelligent SDN security monitoring.

## 4. Proposed Model

**Figure 1:**
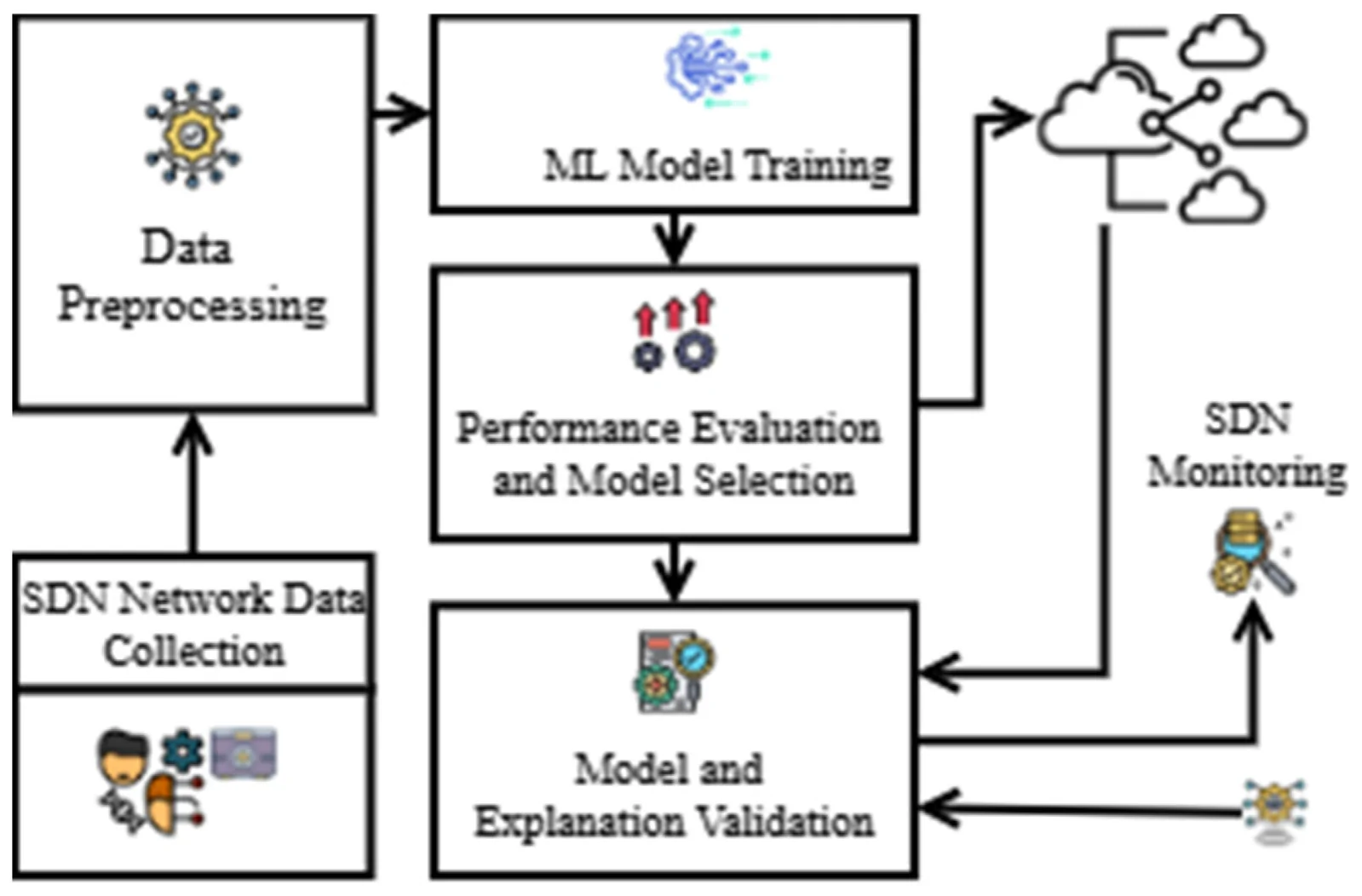
Abstraction of the proposed explainable DDoS attack-detection model.

DDoS attack-detection in SDN networks is often hindered by low adaptability, low generalization capability, high false-alarm rate and poor transparency of decision-making. Traditional signature and rule-based systems are unable to withstand zero-day and obfuscated attacks, and plenty of ML models are black boxes. To address these limitations, this research aims to develop an explainable ML-based DDoS attack-detection framework by combining multiple classifiers, which are equipped with XAI mechanisms to drive accurate and transparent traffic classification results. The framework shown in Figure 1 consists of traffic acquisition, preprocessing, feature preparation, model training and evaluation, optimal model selection and explainability analysis. This approach is structured, allowing for consistent detection of ransomware and also explaining this at global and in-stance levels to assist analysts, build trust and make informed security choices.

### 4.1. Framework Overview

Figure 1 depicts the abstract architecture of the proposed explainable DDoS attack-detection framework in an SDN environment. Network traffic obtained during the data-collection phase is initially captured and then passed to the preprocessing layer, where relevant traffic features are cleaned, transformed, and normalized. The processed data is then passed to the ML-based XAI layer, which consists of multiple machine learning models that classify traffic as legitimate or ransomware and explainability mechanisms, which highlight the features used to make each of these classification decisions. The performance layer assesses the trained models by applying appropriate classification metrics and selects the most reliable model. In the next step, the validation stage is implemented to check the selected model and its explanations before deployment by the SDN infrastructure. The validated detection results enable the continuous monitoring of traffic, identification of threats, and security action based on the analysis of the traffic, and feedback from the network environment is used to evaluate and enhance the framework.

**Figure 2:**
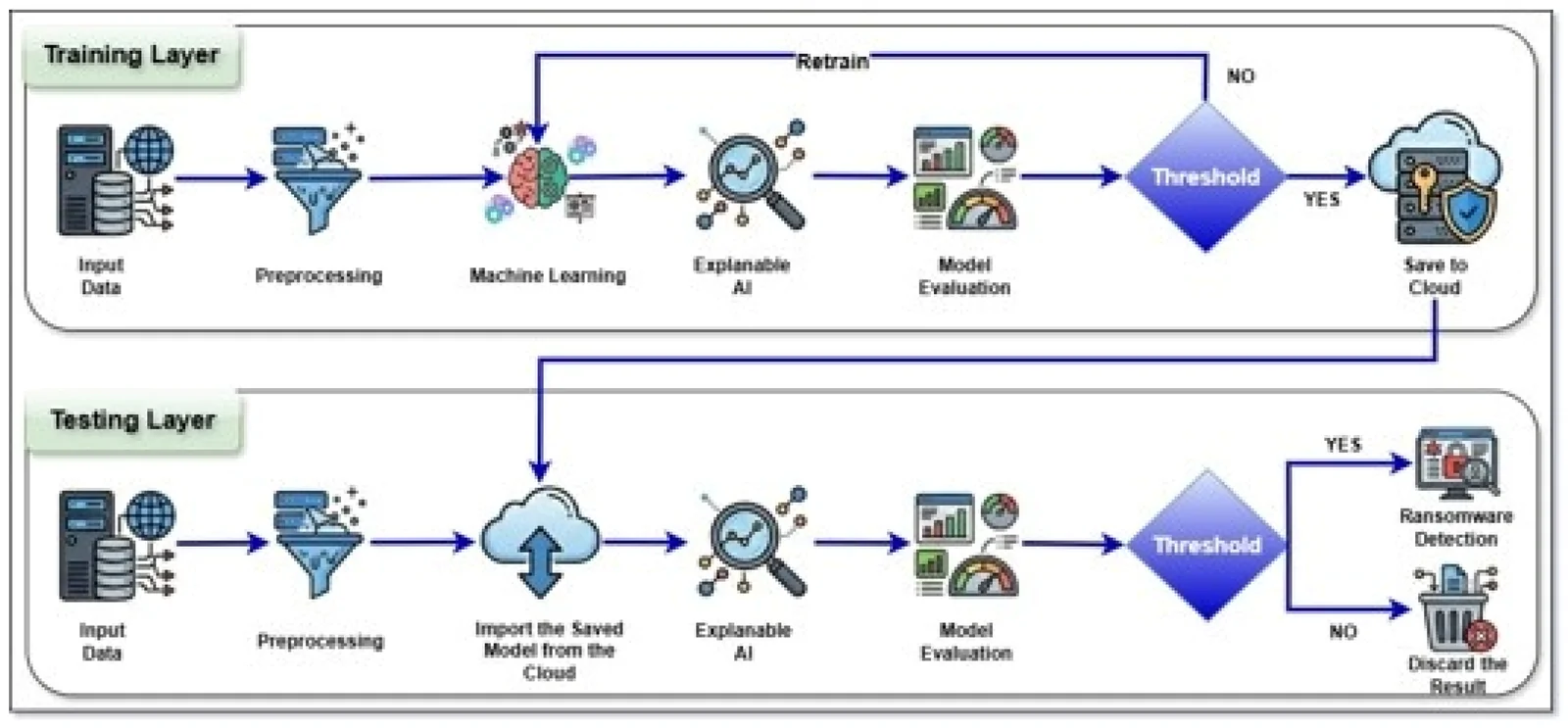
Proposed explainable DDoS attack-detection model.

As illustrated in Figure 2, the proposed framework begins by collecting network-flow records from the centralized SDN controller. The controller provides network-wide visibility and captures statistical information describing packet transmission, flow duration, bandwidth consumption, protocol usage, and communication behavior. The dataset used to implement and evaluate the proposed framework is described separately in Section 4.2.

### 4.2. Dataset Description

The dataset adopted in this study is obtained from [23], and its feature representation is summarized in Table 2. The adopted dataset contains 104,345 SDN flow records comprising benign TCP, UDP, and ICMP traffic and malicious TCP-SYN, UDP-flood, and ICMP-flood traffic. Therefore, the positive class represents DDoS activity, and the present framework is evaluated specifically for flow-based DDoS attack detection rather than endpoint-level DDoS attack detection.

Let the collected dataset be represented as

(1) $$D={\left\{\left(x_{i},y_{i}\right)\right\}}_{i=1}^{N}$$

where N denotes the total number of network-flow observations, $x_{i}\in R^{d}$ represents the feature vector of the i-th observation, and $y_{i}$ denotes its corresponding class label. For binary DDoS attack detection,

(2) $$y_{i}=\left\{\begin{array}{cc} 0, & \mathrm{legitimate}\;\mathrm{traffic}\\ 1, & \mathrm{DDoS}\;\mathrm{attack}\;\mathrm{traffic} \end{array}\right.$$

Since the dataset contains d=24 input attributes, each network-flow instance is expressed as

(3) $$x_{i}=[x_{i1},x_{i2},\ldots ,x_{i24}{]}^{T}$$

Accordingly, the complete feature space can be written in matrix form as

(4) $$X=\left[\begin{array}{cccc} x_{11} & x_{12} & \cdots & x_{1d}\\ x_{21} & x_{22} & \cdots & x_{2d}\\ \vdots & \vdots & \ddots & \vdots \\ x_{N1} & x_{N2} & \cdots & x_{Nd} \end{array}\right]\in R^{N\times d}$$

with the corresponding target vector

(5) $$y=[y_{1},y_{2},\ldots ,y_{N}{]}^{T}$$

The dataset contains 104,345 SDN flow records characterized by packet volume, flow duration, protocol usage, and bandwidth-related features, as summarized in Table 2. The binary target distinguishes benign from DDoS attack traffic.

Accordingly, the detector provides binary flow-level mapping within the standard SDN monitoring and decision workflow: benign flows retain normal handling, whereas suspicious flows identified through protocol, port, traffic-volume, duration, and rate features generate an alert for control-layer policy action. Protocol-specific mitigation rules and standards-conformance testing were outside the scope of this study.

### 4.3. Data Preprocessing

After data acquisition, the feature matrix X is forwarded to the preprocessing layer. This stage removes incomplete, duplicated, inconsistent, and irrelevant observations to obtain a refined dataset

(6) $$D^{\prime }=C(D)$$

where C(·) denotes the cleaning operation.

For a numerical feature $x_{j}$, missing values may be replaced using the median:

(7) $$x_{ij}=\left\{\begin{array}{cc} x_{ij}, & \mathrm{if}\;x_{ij}\;\mathrm{is}\;\mathrm{available}\\ median(x_{j}), & \mathrm{otherwise} \end{array}\right.$$

Duplicate observations are removed according to

(8) $$D^{\prime }=D\setminus \left\{x_{i}:x_{i}=x_{k},\;i\neq k\right\}$$

To prevent features with large numerical ranges from dominating the learning process, each numerical attribute is standardized as

(9) $$z_{ij}=\frac{x_{ij}-\mu_{j}}{\sigma_{j}}$$

where $\mu_{j}$ and $\sigma_{j}$ denote the mean and standard deviation of the j-th feature, respectively. The normalized feature matrix is therefore represented as

(10) $$Z=[z_{ij}{]}_{N\times d}$$

The preprocessed dataset is subsequently divided into training and testing subsets:

(11) $$D^{\prime }=D_{train}\cup D_{test},\;D_{train}\cap D_{test}=\emptyset$$

If the training proportion is denoted by α, then

(12) $$|D_{train}|=\alpha N,\;|D_{test}|=(1-\alpha )N$$

The training subset is used to learn the parameters of the machine learning models, whereas the testing subset is retained for independent performance evaluation. This mathematical preprocessing pipeline improves data consistency, reduces scale-related bias, and prepares reliable inputs for the subsequent ransomware classification and explainability stages.

**Figure 3:**
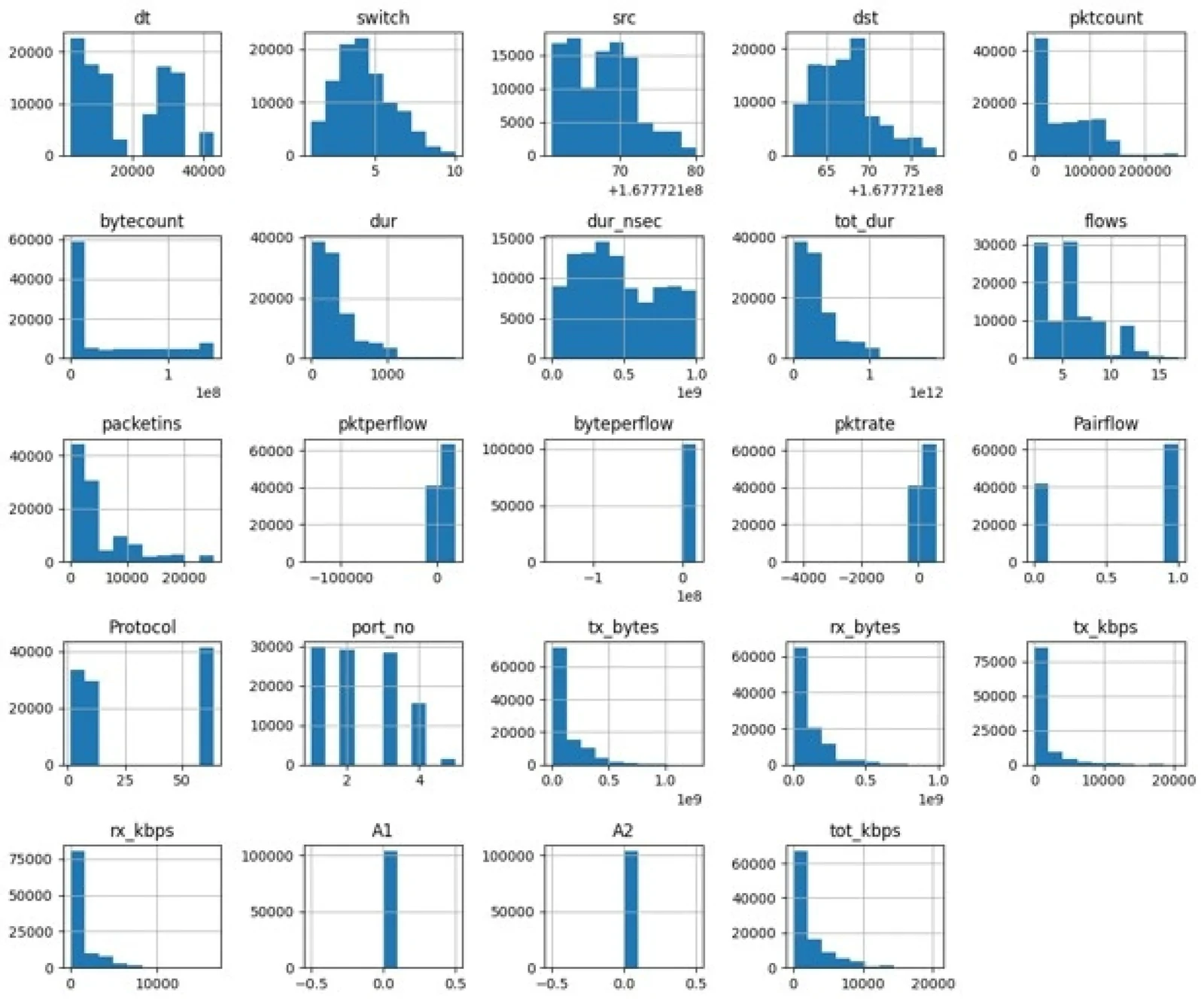
Distribution of network traffic features.

Figure 3 presents the distributions of the network-flow features used for DDoS attack detection. Features such as pktcount, bytecount, and tx_bytes are strongly right-skewed, while time- and rate-based variables show considerable variability and Protocol and port_no form distinct groups. This heterogeneity supports appropriate preprocessing and numerical scaling before model training.

**Figure 4:**
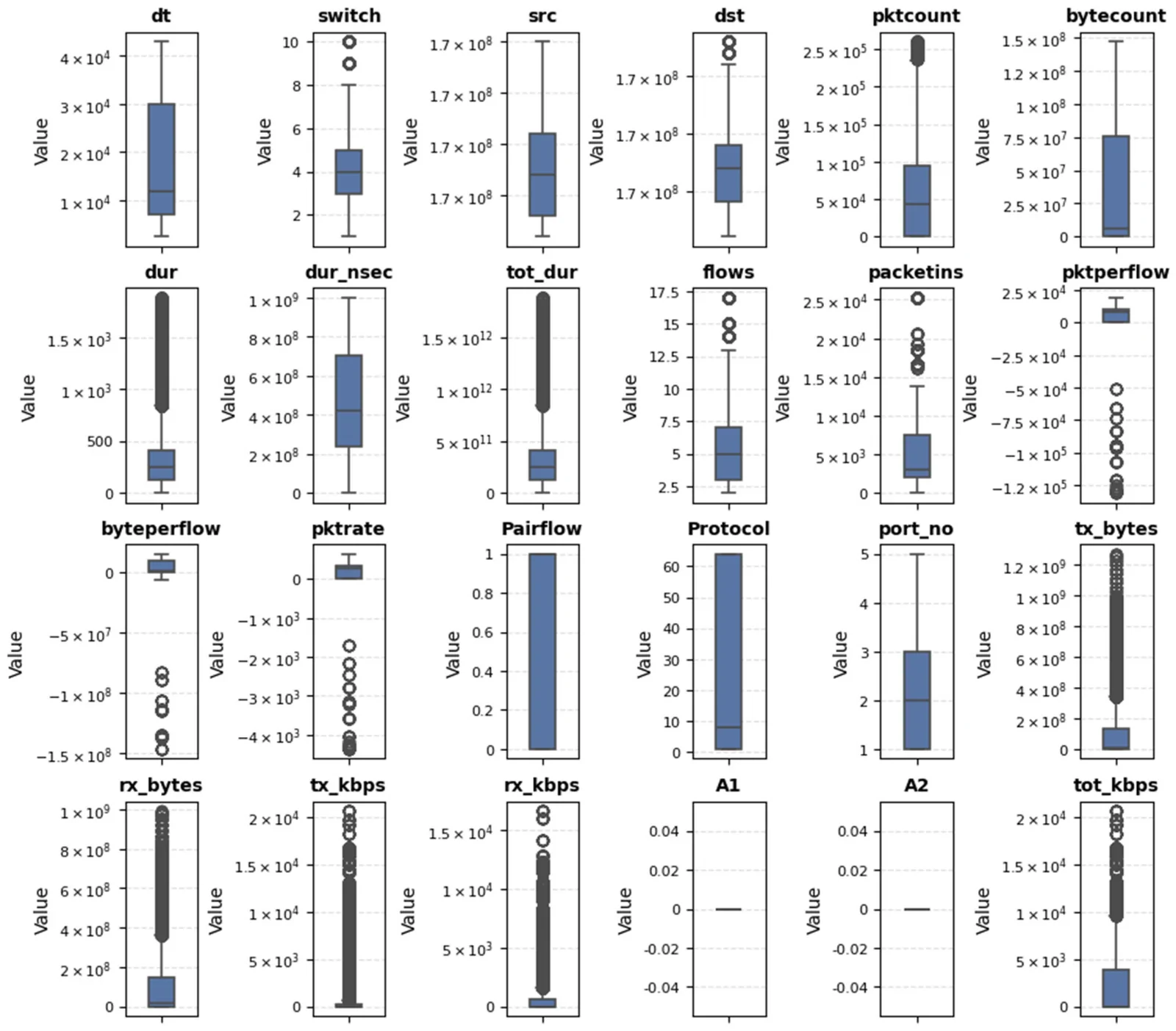
Boxplots of network traffic features.

Figure 4 illustrates the spread and outlier patterns of the network-flow features. Several variables, particularly tot_dur, contain extreme observations that may represent abnormal or malicious traffic rather than data errors. These observations were therefore considered during preprocessing to control scale-related effects without indiscriminately removing potentially informative attack patterns.

**Figure 5:**
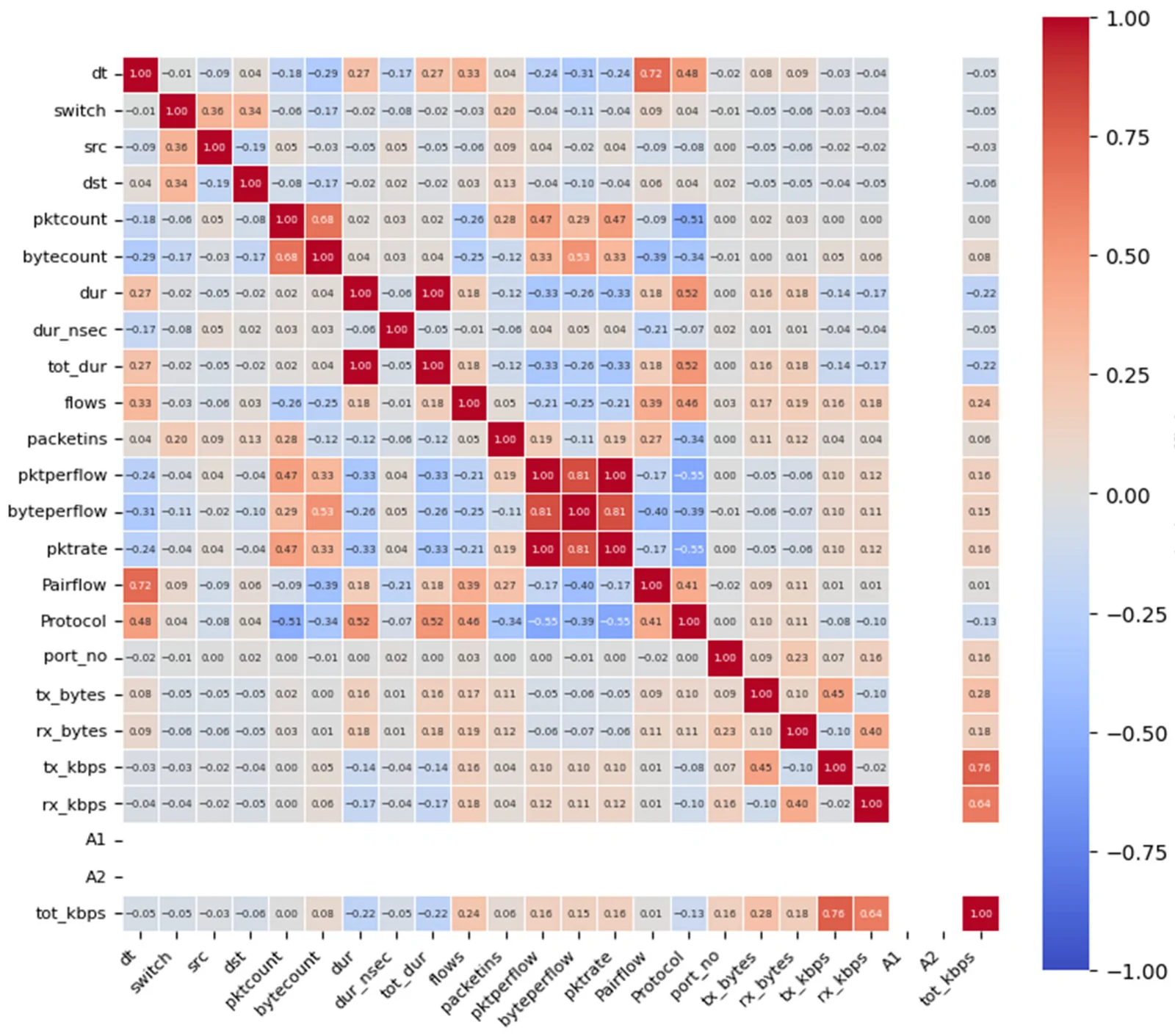
Correlation matrix of network traffic features.

Figure 5 presents the pairwise correlations among the network-flow attributes. Most feature pairs show weak-to-moderate relationships, whereas stronger correlations occur among related traffic-volume, duration, and transmission-rate variables. The analysis consequently helps identify potential redundancy and multicollinearity before DDoS traffic classification.

Accordingly, numerical features were standardized, while extreme observations were retained because they may represent DDoS activity. No feature was removed solely because of correlation; the same feature space was retained for consistent model comparison. Exploratory analysis indicated skewed traffic-volume and duration features, attack-relevant extreme observations, and correlations among related throughput variables. Numerical features were standardized, while extreme observations were retained to avoid removing potentially malicious traffic patterns.

The 104,345 records comprised 63,561 benign and 40,784 DDoS flows. A 70:30 split produced 73,041 training and 31,304 testing instances, consistent with the confusion matrices reported in Table 3.

### 4.4. Machine Learning-Based Ransomware Classification

The preprocessed training set $D_{train}=\{(x_{i},y_{i}){\}}_{i=1}^{N_{tr}}$ is supplied to supervised classifiers, where each model learns a decision function

(13) $$f_{m}:R^{d}\to \{0,1\},m\in \{GB,LR,AdaBoost,GNB\}$$

where $f_{m}(x_{i})=0$ denotes benign traffic and $f_{m}(x_{i})=1$ denotes DDoS attack traffic. For probabilistic models, the predicted ransomware label is determined by

(14) $${\hat{y}}_{i}=\left\{\begin{array}{cc} 1, & P(y_{i}=1|x_{i})\geq \tau \\ 0, & P(y_{i}=1|x_{i})<\tau \end{array}\right.$$

where τ is the classification threshold. The trained models are evaluated using the testing set, and the optimal classifier is selected according to a performance function based on accuracy, precision, recall, F1-score, and miss rate:

(15) $$m^{*}=arg\;\underset{m}{max}\;P(f_{m})$$

As shown in the training layer of Figure 2, retraining is event-driven and triggered when validation performance falls below the acceptance threshold, error rates increase persistently, or sufficient labeled samples of a new DDoS attack become available. New and historical flows are combined, and the updated model is deployed only after meeting the validation criteria. Continuous retraining remains to be evaluated in an online SDN environment.

### 4.5. Explainable Artificial Intelligence Integration

To enhance the transparency of the selected ransomware classifier f(x), the proposed framework integrates both SHapley Additive exPlanations (SHAP) and Local Interpretable Model-Agnostic Explanations (LIMEs). SHAP provides consistent global and local feature-attribution values, whereas LIME approximates the model behavior around an individual traffic instance using an interpretable local surrogate model.

SHAP and LIME were applied without algorithmic modification; their SDN-specific role is to explain controller-level flow features and translate model predictions into analyst-understandable evidence. For SHAP, the contribution of feature j to the prediction of instance $x_{i}$ is defined as

(16) $$\phi_{j}(f,x_{i})=\sum_{S\subseteq F\setminus \{j\}}\frac{|S|!\;(|F|-|S|-1)!}{|F|!}\left[f_{S\cup \{j\}}(x_{i})-f_{S}(x_{i})\right]$$

where F denotes the complete set of input features, S is a subset excluding feature j, and $f_{S}$ represents the model output using the features in S. The prediction for the i-th traffic flow is decomposed as

(17) $$f(x_{i})=\phi_{0}+\sum_{j=1}^{d}\phi_{ij}$$

where $\phi_{0}=E[f(X)]$ is the baseline prediction and $\phi_{ij}$ indicates whether feature j increases or decreases the predicted DDoS attack risk. Global feature importance is obtained by

(18) $$I_{j}^{SHAP}=\frac{1}{N}\sum_{i=1}^{N}|\phi_{ij}|$$

Thus, features with larger $I_{j}^{SHAP}$ values exert a stronger overall influence on DDoS attack detection.

LIME explains an individual prediction by generating perturbed samples around $x_{i}$ and fitting a simple interpretable model $g_{i}$. The local explanation is obtained through

(19) $$g_{i}^{*}=arg\;\underset{g\in G}{min}\left[L\left(f,g,\pi_{x_{i}}\right)+\Omega (g)\right]$$

where G is the class of interpretable surrogate models, L measures the local difference between the original classifier f and surrogate g, $\pi_{x_{i}}$ assigns greater weights to samples closer to $x_{i}$, and Ω(g) penalizes model complexity. For a weighted linear surrogate, the optimization becomes

(20) $$\beta_{i}^{*}=arg\;\underset{\beta }{min}\sum_{k=1}^{K}\pi_{x_{i}}(z_{k}){\left[f(z_{k})-\left(\beta_{0}+\beta^{T}z_{k}\right)\right]}^{2}+\lambda ∥\beta {∥}_{1}$$

where $z_{k}$ denotes a perturbed traffic sample, β represents local feature weights, and λ controls explanation sparsity. The proximity function may be expressed as

(21) $$\pi_{x_{i}}(z_{k})=exp(-\frac{∥x_{i}-z_{k}{∥}_{2}^{2}}{\sigma^{2}})$$

The resulting coefficients $\beta_{j}$ indicate the direction and magnitude of each feature’s influence on the selected traffic prediction. By combining SHAP-based global and local attribution with LIME-based instance-level explanations, the proposed framework identifies influential SDN traffic characteristics, validates DDoS attack alerts, and improves analyst confidence in automated detection decisions.

### 4.6. Testing and Decision Phase

During testing, each incoming network-flow instance is first passed through the same preprocessing pipeline used during training to ensure feature consistency. The optimized model is then retrieved from cloud storage and applied to the processed input to generate a DDoS attack probability,

(22) $${\hat{p}}_{i}=f^{*}(x_{i})$$

The final decision is determined using a predefined threshold τ:

(23) $${\hat{y}}_{i}=\left\{\begin{array}{cc} 1, & {\hat{p}}_{i}\geq \tau \;\left(\mathrm{ransomware}\;\mathrm{detected}\right)\\ 0, & {\hat{p}}_{i}<\tau \;\left(\mathrm{benign}\;\mathrm{traffic}\right) \end{array}\right.$$

The prediction is subsequently interpreted using SHAP and LIME to identify the traffic features that contributed most strongly to the decision. A positive decision triggers a DDoS attack alert and security response, whereas a negative decision allows the traffic to continue as legitimate communication.

## 5. Results

In SDN, detecting ransomware is difficult, given that malicious traffic may be hard to distinguish from normal traffic, can change quickly, and can exploit encrypted or obfuscated channels. To solve these challenges, the proposed explainable machine learning model was implemented in a Google Colab Python 3.12.13-based environment and tested with various classification methods. The preprocessed dataset was split into 70% training and 30% testing data, with the training data used to train the DDoS attack traffic pattern and the testing data used to evaluate the model’s generalization on unseen data. The accuracy, precision, sensitivity, specificity, F1-score, negative predictive value, false-positive rate, false-negative rate, balanced accuracy, Matthews correlation coefficient, Cohen’s kappa, and ROC-AUC were used to assess the performance of each classifier. These measures provide a comprehensive assessment of detection reliability, missed attacks, false alarms, and class-wise predictive capability.

Let TP, TN, FP, and FN denote true positives, true negatives, false positives, and false negatives, respectively. The evaluation metrics are defined as follows:

(24) $$\mathrm{Accuracy}=\frac{TP+TN}{TP+TN+FP+FN}$$

(25) $$\mathrm{Precision}=\frac{TP}{TP+FP}$$

(26) $$\mathrm{Sensitivity}=\mathrm{Recall}=\frac{TP}{TP+FN}$$

(27) $$\mathrm{Specificity}=\frac{TN}{TN+FP}$$

(28) $$F1\text{-}\mathrm{score}=\frac{2\times \mathrm{Precision}\times \mathrm{Sensitivity}}{\mathrm{Precision}+\mathrm{Sensitivity}}=\frac{2TP}{2TP+FP+FN}$$

(29) $$\mathrm{Negative}\;\mathrm{Predictive}\;\mathrm{Value}=\frac{TN}{TN+FN}$$

(30) $$\mathrm{False}\;\mathrm{Positive}\;\mathrm{Rate}=\frac{FP}{FP+TN}=1-\mathrm{Specificity}$$

(31) $$\mathrm{False}\;\mathrm{Negative}\;\mathrm{Rate}=\mathrm{Miss}\;\mathrm{Rate}=\frac{FN}{FN+TP}=1-\mathrm{Sensitivity}$$

(32) $$\mathrm{Balanced}\;\mathrm{Accuracy}=\frac{\mathrm{Sensitivity}+\mathrm{Specificity}}{2}$$

The Matthews correlation coefficient is calculated as

(33) $$\mathrm{MCC}=\frac{TP\times TN-FP\times FN}{\sqrt{\left(TP+FP)(TP+FN)(TN+FP)(TN+FN\right)}}$$

Cohen’s kappa is expressed as

(34) $$\kappa =\frac{p_{o}-p_{e}}{1-p_{e}}$$

where $p_{o}$ represents the observed agreement and $p_{e}$ denotes the agreement expected by chance. The ROC-AUC measures the model’s ability to distinguish DDoS attack traffic from benign traffic across different classification thresholds, where a value closer to 1 indicates stronger discriminatory performance.

Table 3 shows that Gradient Boosting achieved the most reliable and balanced classification performance, correctly identifying 19,053 benign and 12,211 ransomware test instances while producing only 16 false positives and 24 false negatives. AdaBoost detected most DDoS attack samples but generated a comparatively high number of false alarms, with 3214 benign flows incorrectly classified as malicious. Logistic Regression showed weaker discrimination, recording 4498 false positives and 2669 missed attacks, whereas Gaussian Naive Bayes produced the poorest results with 5864 false positives and 4893 false negatives. Overall, Gradient Boosting demonstrated the best trade-off between DDoS attack detection, false-alarm control, and generalization to unseen SDN traffic.

The 16 false positives represent approximately 0.08% of benign test flows and may cause unnecessary blocking, whereas the 24 false negatives represent approximately 0.20% of DDoS flows and indicate missed attacks. Their specific feature-level causes were not separately evaluated; future work will apply SHAP and LIME to these misclassified cases.

Table 4 confirms that Gradient Boosting delivered the strongest and most consistent performance across both training and testing sets. It achieved 99.88% training accuracy and 99.87% testing accuracy, with sensitivity, specificity, precision, and F1-score all approximately 99.8% or higher. Its ROC-AUC of 1.0000, MCC above 0.997, and extremely low training and testing error rates of 0.12% and 0.13%, respectively, further demonstrate excellent class discrimination and generalization. AdaBoost ranked second, attaining approximately 89% accuracy and very high sensitivity above 99%, but its lower specificity and precision indicate a greater tendency to misclassify benign traffic as DDoS attack traffic. Logistic Regression produced only moderate performance, with nearly 77% accuracy and training and testing error rates of 23.00% and 22.89%, respectively, whereas Gaussian Naive Bayes performed weakest, achieving about 65.6% accuracy with error rates of 34.30% and 34.36%. Overall, the close agreement between Gradient Boosting’s training and testing results indicates stable learning with minimal overfitting, making it the most suitable model for reliable and explainable DDoS attack detection in SDN environments.

Based on this superior performance, SHAP and LIME were integrated with the Gradient Boosting model to provide global feature importance and instance-level explanations for its DDoS attack predictions, thereby establishing complementary global and local interpretability.

**Figure 6:**
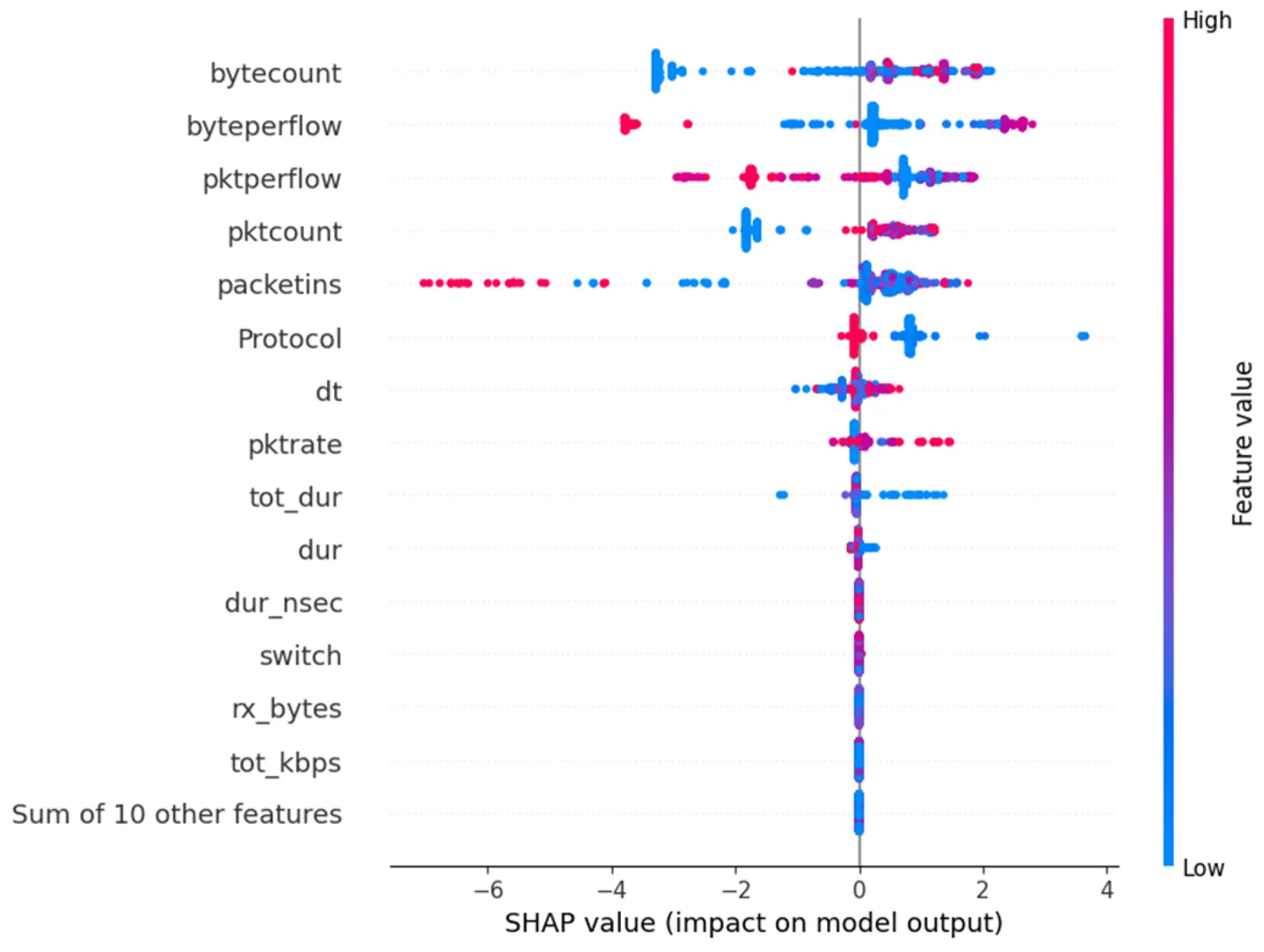
SHAP summary plot of the Gradient Boosting model for DDoS attack detection.

The SHAP summary plot in Figure 6 shows that bytecount, byteperflow, pktperflow, pktcount, and packetins are the most influential features in the Gradient Boosting predictions. bytecount exhibits SHAP values ranging approximately from −3.2 to +2.2, while byteperflow spans nearly −3.8 to +2.8, indicating strong bidirectional effects on the predicted class. packetins produces the largest negative impact, reaching about −7.0, whereas Protocol shows the strongest positive contribution, approaching +3.8 for some observations. Features such as dur_nsec, switch, rx_bytes, and tot_kbps remain concentrated around zero, indicating limited influence on the model output. Overall, positive SHAP values push predictions toward the DDoS attack class, whereas negative values support benign classification; red and blue points represent high and low feature values, respectively. Thus, SHAP provides global explainability by revealing each feature’s relative importance, contribution direction, and effect magnitude across traffic instances.

**Figure 7:**
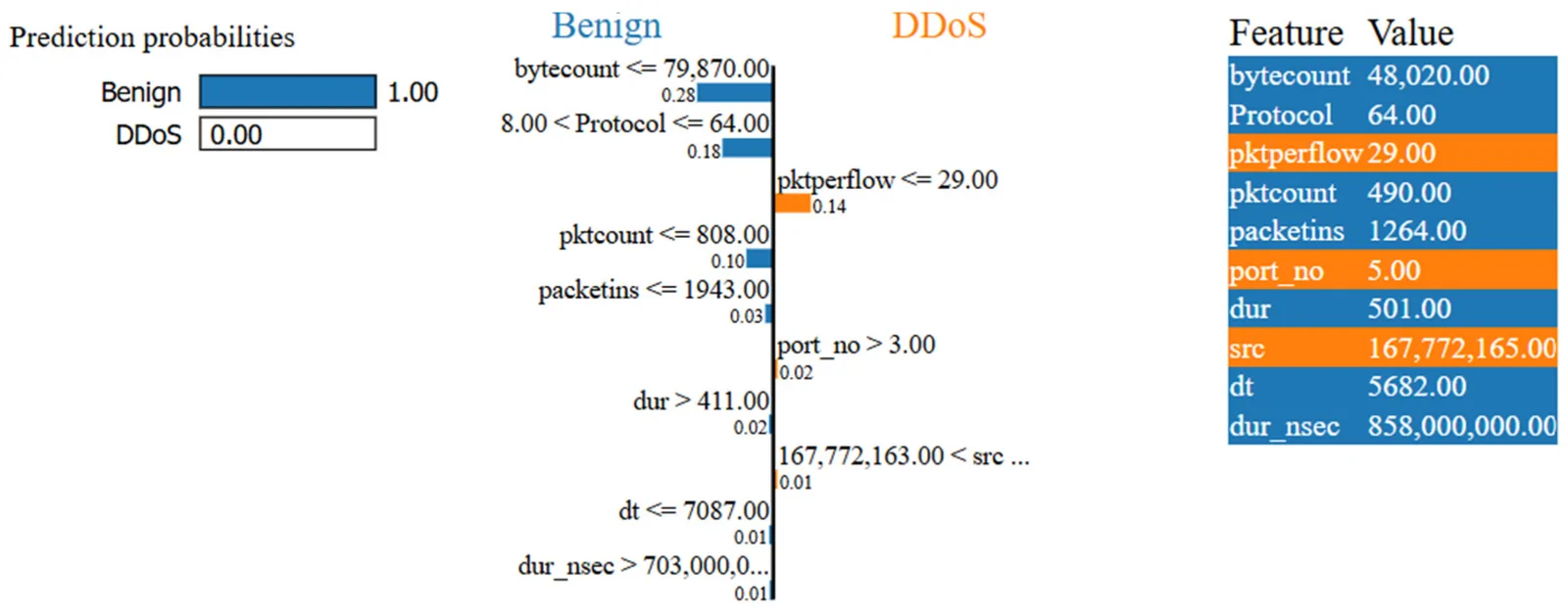
LIME explanation of a benign traffic prediction generated by the Gradient Boosting model.

The LIME explanation in Figure 7 shows that the selected network-flow instance was classified as benign with a probability of 1.00, while the DDoS attack probability was approximately 0.00. The strongest factors supporting the benign decision were bytecount = 79,870, Protocol = 64, pktcount = 490, packetins = 1264, and dur = 501, which collectively shifted the prediction toward the benign class. In contrast, pktperflow = 29 and port_no = 5 provided relatively small support for the ransomware class, with local contribution weights of approximately 0.14 and 0.02, respectively. Overall, the substantially larger combined benign contributions outweighed the limited ransomware evidence, confirming that the model’s decision was primarily driven by the observed traffic-volume, protocol, packet-count, and duration characteristics. Thus, LIME provides instance-level explainability by linking the final benign probability to the observed feature values and their corresponding local contribution weights.

**Figure 8:**
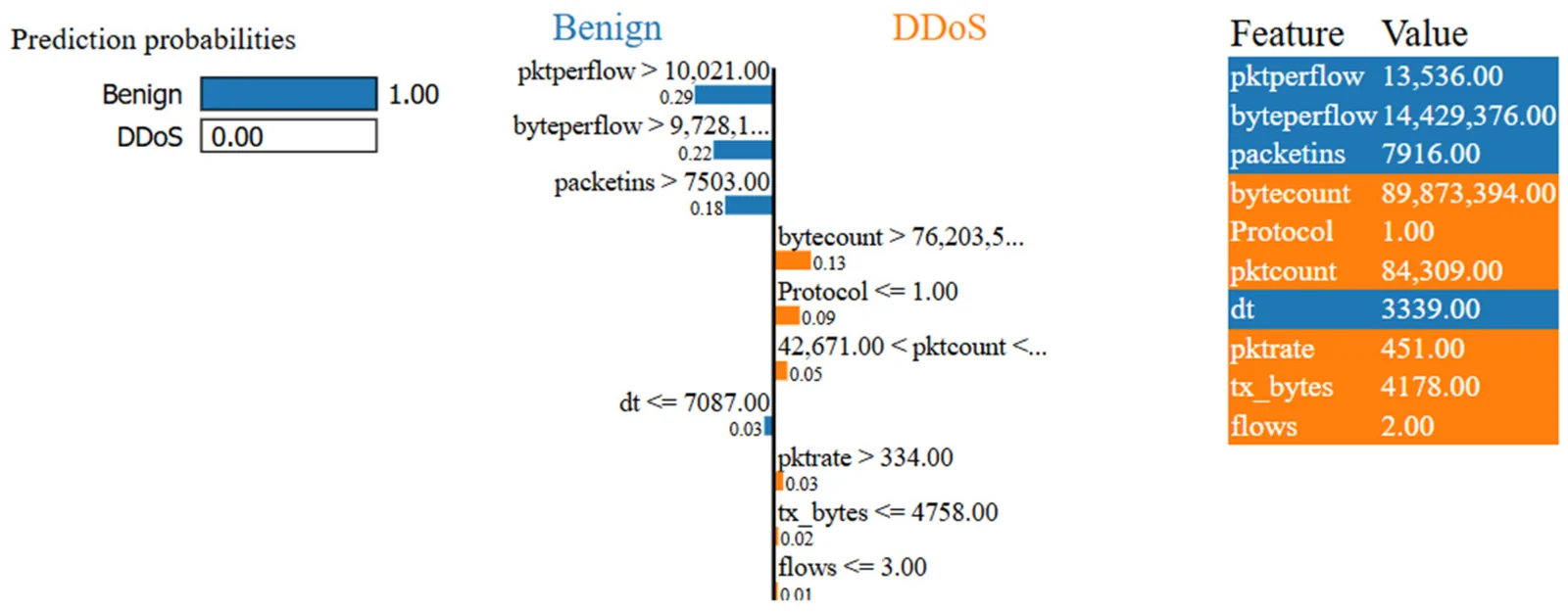
LIME explanation of the second benign traffic prediction generated by the Gradient Boosting model.

The selected instance in Figure 8 was classified as benign with a probability of 1.00, while the DDoS attack probability remained close to 0.00. The strongest benign contributions were associated with pktperflow > 10,021, byteperflow > 972,881, and packetins > 7503, with approximate local weights of 0.29, 0.22, and 0.18, respectively. In contrast, high bytecount, Protocol = 1, elevated pktcount, pktrate, tx_bytes, and flows provided comparatively weaker support toward the ransomware class. However, their combined influence was insufficient to outweigh the dominant benign evidence. Overall, the explanation indicates that packet-flow intensity, byte-flow volume, and packet-in activity were the principal factors driving the model toward a benign classification. The recurrence of pktperflow, byteperflow, and packetins across the global SHAP and local LIME results further demonstrates qualitative consistency between the two explanation methods.

SHAP and LIME evidence from key flow features can help analysts validate DDoS alerts and initiate rate limiting or flow blocking. To reduce overhead, global SHAP can be computed offline and local explanations generated only for flagged flows; explanation latency was not evaluated.

The proposed Gradient Boosting model achieved the highest accuracy of **99.87%** and the lowest miss rate of **0.13%** among the compared studies. It also integrates SHAP and LIME, providing both strong detection performance and transparent decision interpretation. Since the studies used different datasets and experimental settings, the comparison should be considered contextual rather than direct.

Table 5 also includes modern CNN, BiLSTM, and RTS-DELM approaches; however, these comparisons remain contextual because different datasets and protocols were used.

## 6. Conclusions

Reliable DDoS attack detection is increasingly essential in SDN environments because delayed or inaccurate identification can lead to data encryption, service disruption, lateral propagation, and compromise of critical network resources. This challenge is intensified by rapidly evolving ransomware variants, encrypted communication, dynamic traffic behavior, and the growing similarity between malicious and legitimate flows. Traditional signature- and rule-based mechanisms remain effective mainly against known attacks, while many machine learning approaches provide strong predictions without sufficient transparency, limiting analyst trust and practical deployment. To address these limitations, this research developed an explainable machine learning framework that combines multiple classifiers with SHAP- and LIME-based interpretation. Among the evaluated models, Gradient Boosting achieved the strongest and most consistent performance, attaining 99.88% training accuracy, 99.87% testing accuracy, a ROC-AUC of 1.000, and a testing miss rate of only 0.13%. It correctly identified 19,053 benign and 12,211 ransomware test instances, with only 16 false positives and 24 false negatives. SHAP further identified bytecount, byteperflow, pktperflow, pktcount, and packetins as the most influential global features, whereas LIME provided understandable explanations for individual predictions. Overall, the proposed framework improves not only detection accuracy but also transparency, analyst confidence, and decision support. These positive outcomes demonstrate its potential as a trustworthy and operationally effective solution for intelligent ransomware monitoring in SDN-based infrastructures.

### 6.1. Limitations

The study was evaluated using a single SDN dataset and a fixed train–test split. Its generalization across different network environments, DDoS attack types, traffic distributions, and real-time deployment conditions therefore requires further validation. The current framework also focuses on binary classification and does not assess explanation latency, concept drift, adversarial robustness or hardware-specific computational costs, including inference latency, memory usage, and explanation overhead. The offline model was not evaluated for adversarial robustness, concept drift, or real-time computational cost.

### 6.2. Future Work

Future work will evaluate the framework using multiple real-world datasets, cross-validation, and cross-dataset testing. The model can also be extended toward DDoS attack-type classification, online learning, lightweight explainability, adversarial defenses, and automated SDN-controller responses. Computational time, memory consumption, and XAI overhead will also be evaluated for real-time deployment.

## Figures and Tables

**Table 1:** Comparative analysis of related studies.

Ref.	Method	Objective	Achievement	XAI	Strength	Limitation
[13]	SDN, PFE, RF	Detect HTTPS ransomware	Detection > 0.86; FNR < 0.11	✗	No payload inspection	Single model; no explainability
[14]	ML, DL, FL, entropy	Review ransomware detection	Identified adaptive methods	✓	Broad recent review	Data imbalance; high cost
[15]	ML and DL	Detect zero-day ransomware	Captured complex patterns	✗	Advanced detection coverage	Weak generalization; costly
[16]	ML and DL	Proactive ransomware detection	Supported unseen-threat detection	✗	Focus on early detection	Limited adaptability
[17]	ML, GNN, LLM, XAI	Review modern ML-IDS	Covered advanced IDS methods	✓	Broad and current scope	Scalability and trust issues
[18]	SVM, RF, CNN, GAN	Review AI-based IDS	Compared models and tools	✓	Practical IDS taxonomy	Bias and deployment issues
[19]	Anomaly-based ML	Detect unknown intrusions	Improved adaptability	✗	Supports novel attacks	False alarms; weak scalability
[20]	Feature processing, ML	Review IDS datasets	Compared major datasets	✗	Strong dataset analysis	Imbalance and redundancy
[21]	PCA, KNN, RF, DT, SVM	Real-time intrusion detection	High accuracy; Flask deployment	✗	Multi-model evaluation	No XAI; limited validation
Proposed Work		Multiple ML models, Gradient Boosting, XAI	Explainable ransomware detection in SDN|99.72% training and 99.65% testing accuracy	✓	High accuracy and transparent decisions	Requires validation on larger real-world datasets

**Table 2:** Description of the SDN network-flow dataset [23].

Sr. No.	Feature	Datatype
0	dt	int64
1	switch	int64
2	src	int64
3	dst	int64
4	pktcount	int64
5	bytecount	int64
6	dur	int64
7	dur_nsec	int64
8	tot_dur	float64
9	flows	int64
10	packetins	int64
11	pktperflow	int64
12	byteperflow	int64
13	pktrate	int64
14	Pairflow	int64
15	Protocol	int64
16	port_no	int64
17	tx_bytes	int64
18	rx_bytes	int64
19	tx_kbps	int64
20	rx_kbps	float64
21	A1	int64
22	A2	int64
23	tot_kbps	float64

**Table 3:** Combined training and testing confusion-matrix values of the proposed model.

Model	Train TN	Train FP	Train FN	Train TP	Test TN	Test FP	Test FN	Test TP
Gradient Boosting	44,448	44	46	28,503	19,053	16	24	12,211
Logistic Regression	34,036	10,456	6343	22,206	14,571	4498	2669	9566
AdaBoost	36,782	7710	226	28,323	15,855	3214	92	12,143
Gaussian Naive Bayes	30,820	13,672	11,382	17,167	13,205	5864	4893	7342

**Table 4:** Combined training and testing performance of the proposed model.

Model	Dataset	Accuracy	Sensitivity	Specificity	Precision	F1-Score	ROC-AUC	MCC	Miss Rate
Gradient Boosting	Train	0.9988	0.9984	0.9990	0.9985	0.9984	1.0000	0.9974	0.0012
Gradient Boosting	Test	0.9987	0.9980	0.9992	0.9987	0.9984	1.0000	0.9973	0.0013
Logistic Regression	Train	0.7700	0.7778	0.7650	0.6799	0.7256	0.8427	0.5327	0.23
Logistic Regression	Test	0.7711	0.7819	0.7641	0.6802	0.7275	0.8441	0.5356	0.2289
AdaBoost	Train	0.8913	0.9921	0.8267	0.7860	0.8771	0.9677	0.7991	0.1087
AdaBoost	Test	0.8944	0.9925	0.8315	0.7907	0.8802	0.9684	0.8042	0.1056
Gaussian Naive Bayes	Train	0.6570	0.6013	0.6927	0.5567	0.5781	0.7373	0.2905	0.343
Gaussian Naive Bayes	Test	0.6564	0.6001	0.6925	0.5560	0.5772	0.7379	0.2891	0.3436

**Table 5:** Contextual comparison of the proposed DDoS attack-detection model with prior studies.

Reference	Detection Model	XAI	Accuracy (%)	Miss Rate (%)
Gadze et al., 2021 [24]	CNN	✗	66	34
Ahmed et al., 2022 [25]	Random Forest	✗	95	5
Haider et al., 2021 [26]	RTS-DELM	✗	92.7	7.3
Nazir et al., 2021 [27]	Random Forest	✗	83.12	16.88
Imrana et al., 2021 [28]	Bidirectional LSTM	✗	94.26	5.74
Mane et al., 2021 [29]	Multilayer Perceptron	✓	82.00	18.00
Farooq et al., 2023 [30]	Fused ML Model	✗	95.18	4.82
D’hooge et al., 2022 [31]	Random Forest	✗	91.00	9.00
Kasongo et al., 2020 [32]	Decision Tree	✗	90.85	9.15
Proposed Work	Gradient Boosting with SHAP and LIME	✓	99.87	0.13

## Data Availability

The SDN-DDoS dataset used in this study is publicly available through the Kaggle notebook “DDoS Attack Detection Classification a03d6b,” curated by Atul Kumar. The exact dataset snapshot is available in the notebook’s Input section, while the associated preprocessing code is provided within the same notebook (accessed on 28 January 2026).
